# Feasibility, Safety, and Effectiveness of Telerehabilitation in Mild-to-Moderate Parkinson's Disease

**DOI:** 10.3389/fneur.2022.909197

**Published:** 2022-06-16

**Authors:** Edoardo Bianchini, Camilla Onelli, Carmen Morabito, Marika Alborghetti, Domiziana Rinaldi, Paolo Anibaldi, Adriano Marcolongo, Marco Salvetti, Francesco E. Pontieri

**Affiliations:** ^1^Department of Neuroscience, Mental Health and Sensory Organs (NESMOS), Sapienza University of Rome, Rome, Italy; ^2^Sant'Andrea University Hospital, Rome, Italy; ^3^Istituto di Ricovero e Cura a Carattere Scientifico (IRCCS) Fondazione Santa Lucia, Rome, Italy; ^4^Istituto di Ricovero e Cura a Carattere Scientifico (IRCCS) Istituto Neurologico Mediterraneo (INM) Neuromed, Pozzilli, Italy

**Keywords:** neurorehabilitation, Parkinson's disease, physiotherapy, remote treatment, telehealth, telemedicine, telerehabilitation

## Abstract

**Introduction:**

Parkinson's disease (PD) patients frequently engage in rehabilitation to ameliorate symptoms. During the Coronavirus disease 2019 (COVID-19) pandemic, access to rehabilitation programs has been markedly limited, consequently, telerehabilitation gained popularity. In this prospective, open-label, and pilot study, we aimed to investigate feasibility, safety, and efficacy of telerehabilitation in mild-to-moderate PD patients.

**Materials and Methods:**

Twenty-three PD patients, with Hoehn and Yahr stage <3, without gait disturbances or dementia and capable of using the televisit platform, were recruited for a 5-week telerehabilitation program, consisting of 1 remote visit with a therapist and a minimum of two sessions of >30-min of self-conducted exercises per week. Patients received video tutorials of exercises and were asked to keep a diary of sessions. At baseline (T0), at the end of the intervention (T1), and 1 month after the end of treatment (T2), patients were remotely assessed with MDS-UPDRS part I-III, PDQ-39, Functional Independence Measure (FIM), and Frontal Assessment Battery scales, respectively. Acceptable compliance to the program was defined as >60% matching of frequency and duration of sessions, whereas optimal compliance was set at >80% matching.

**Results:**

The dropout rate was 0%. Over 85% of patients reached acceptable adherence cut-off and around 70% reached optimal one. No adverse events were reported during sessions. The repeated measure analysis of variance (rANOVA) showed a significant effect of factor “time” for MDS-UPDRS-III (*p* < 0.0001) with a mean reduction of 4.217 points between T0 and T1 and return to baseline at T2. No significant effect was found for other outcome measures.

**Conclusion:**

Our findings demonstrate that telerehabilitation is safe, feasible, and effective on motor symptoms in mild-to-moderate PD patients.

## Introduction

Parkinson's disease (PD) is the second most common neurodegenerative disease in terms of prevalence and burden of disability (1). The primary symptoms of PD include bradykinesia, rigidity, and resting tremor. Additional and more disabling motor symptoms, such as postural instability and gait disturbances, frequently occur with disease progression and carry heavy impact on independence and quality of life (QoL) (2, 3). Moreover, PD patients may experience a variety of non-motor symptoms (NMS), such as sensory alterations, dysautonomia, sleep disturbances, mood disorders, and cognitive impairment, which may precede the motor onset or arise along disease course, and further deteriorate the QoL of patients (4). The management of PD relies mostly on symptomatic pharmacological therapy with L-Dopa or other dopaminergic agents (5). Several drugs are available for treating NMS as well (6). However, even with optimal pharmacological management, most PD patients engage in rehabilitation to reduce disability in daily activities. Physiotherapy is the most widely used rehabilitation approach and has the most solid result evidence, in particular on motor symptoms of PD (5–7). In this respect, the European Physiotherapy Guidelines for PD offer a useful tool for clinicians to evaluate patients and refer them to physiotherapists. Moreover, these guidelines represent the evidence-based supports to physiotherapists for identifying treatment goals and intervention strategies tailored to the management of disease staging and severity (8).

The recent Coronavirus disease 2019 (COVID-19) pandemics widely disrupted most of our daily life aspects and forced administrations to lockdown and strict social distancing measures. This had a heavy impact on the healthcare systems as well, with chronic disease patients being the most affected. Indeed, reports of worsening of some NMS, in particular anxiety, in PD patients have accumulated in the last 2 years (9–18). This was associated mostly with difficulties in accessing clinical services and medications (19), reduction of physical activity, and inability to access rehabilitation clinics (20), with up to 88% of patients reporting the interruption of physiotherapy during lockdown (16). To overcome these limitations, a transition from in-person to remote visits has been supported by several PD centers for implementing telemedicine and telehealth management of PD patients (21–24).

Telemedicine represents an interface in a virtual patient–physician relationship to provide primary and secondary care for a variety of neurological disorders (25). With respect to PD, telemedicine has been applied to assist remote management of devices for advanced therapies, teleconsultation, telerehabilitation, and monitoring of motor and non-motor parameters in an ecologically valid environment (26). In the field of rehabilitation, the call for implementing telemedicine instruments to ensure continuity in the management of neurological patients was strong (27–31). In Italy, the Italian Society for the Neurological Rehabilitation published a guideline containing urgent measures to face limitations imposed by severe acute respiratory syndrome coronavirus 2 (SARS-CoV2)-pandemics, including the use of remote assessments and management solutions (32). The remote administration of physiotherapy in PD patients is rather challenging, and the feasibility of treatment is hampered by the fear of adverse events (AEs), particularly falls without the possibility of prompt intervention by the operator. Despite these concerns, there is growing evidence in favor of the efficacy of telerehabilitation to sustain physical activity, mobility, and emotional wellbeing (23, 29, 33–39). Most reports dated before the COVID-19 pandemics were focused on cognitive training, speech therapy, and dance therapy in small cohorts of patients affected by different neurological disorders. In the present study, we sought to investigate the feasibility, safety, and efficacy of telerehabilitation in mild-to-moderate PD patients. The program was originally designed and carried out during the lockdown due to the COVID-19 pandemics in Italy, then maintained after the reopening of rehabilitation facilities.

## Materials and Methods

This was a prospective, open-label pilot study, aimed to investigate the feasibility, safety, and efficacy of telerehabilitation in mild-to-moderate PD patients. The study was performed in accordance with the ethical standards as laid down in the 1964 Declaration of Helsinki and its later amendments. Approval was granted by the Local Ethical Committee of the Sapienza University of Rome. Data collection and processing followed the current European regulation for data protection. Patients with PD, referring to our Movement Disorder Outpatient Service in the period between January 2020 and August 2021 were screened for enrollment with a 1:10 ratio according to the visit schedule. The inclusion criteria were: (i) diagnosis of idiopathic PD according to the MDS criteria (40); (ii) disease stage <3 according to the modified Hoehn and Yahr (H&Y) scale (41); (iii) stable antiparkinsonian treatment in the previous 3 months; (iv) availability of technical instruments for remote video-call (tablet, laptop, or computer/webcam) and ability to use them by patients and/or caregiver; (v) availability and motivation of patients to participate to a 5-weeks telerehabilitation program; and (vi) attendance of a caregiver during remote and self-conducted sessions for patients with H&Y score >1. The exclusion criteria were: (i) contraindications to rehabilitation treatment; (ii) patients already undergoing rehabilitation treatment; (iii) co-morbidity with non-stabilized major medical illnesses; (iv) cognitive impairment as defined by a Mini-Mental State Examination (MMSE) score <24; and (v) presence of freezing of gait (FOG).

Enrolled patients matching inclusion and exclusion criteria underwent a 5-week telerehabilitation program consisting of a remote session with a physiotherapist once weekly and at least two self-conducted sessions per week. In the 1st week of the treatment, an additional assisted remote session was scheduled for further training and exercise feedback. Moreover, patients had free access to video tutorials, showing the exercises performed with physiotherapists and were instructed to exercise at least twice weekly with a minimum of 30 min for each session. Areas of intervention included general mobility, static, and dynamic balance, coordination, dexterity, postural transitions, and facial mobility. Mobility and postural transition exercises focused mainly on sit-to-stand and lying mobility to address in-bed turning difficulties. A number of exercises ranging from 8 to 12, for duration of 40–60 min were included in each session depending on the patients' condition, functional demands, and reported difficulties. Examples of video tutorials are available in the Supplementary Material.

To evaluate compliance, patients were instructed to keep a diary of self-conducted sessions. Patients were evaluated before treatment (T0), at the end of the 5-week treatment program (T1) and 1 month after the end of treatment (T2). All evaluations were performed remotely on a digital platform for telemedicine freely available by Regione Lazio, named “Salute Digitale” (42). The platform consists of an easy-to-access audio/video remote conference call interface based on the open-source set Jitsi Meet. A unique room for teleconsultation is generated by the healthcare provider and the private link for participation is communicated to the patient. The teleconsultation room is canceled automatically at the end of the call. The platform is compliant with GDPR and current regulations for web and software privacy and security.

The primary outcome measures of the present study were feasibility and safety of telerehabilitation. To assess them, we investigated three variables: dropout rate, adherence to the program, and occurrence of AEs. Dropout rate was defined as the rate of patients who did not complete the study from enrollment to post-training evaluation. The *a priori* criterion for adherence was set at a 20% dropout rate. Patient adherence to the telerehabilitation program was defined as the rate of training sessions matching frequency (≥3 sessions per week) and duration (≥30 min). This was considered acceptable for at least 60% and optimal for at least 80% rate, respectively. Falls during the telerehabilitation program were considered the primary AEs. The *a priori* criterion was set at 0 falls. Moreover, any other possible AE occurring during the training program was recorded. Six secondary outcome measures were collected to evaluate the patients' status.

**Figure 1:**
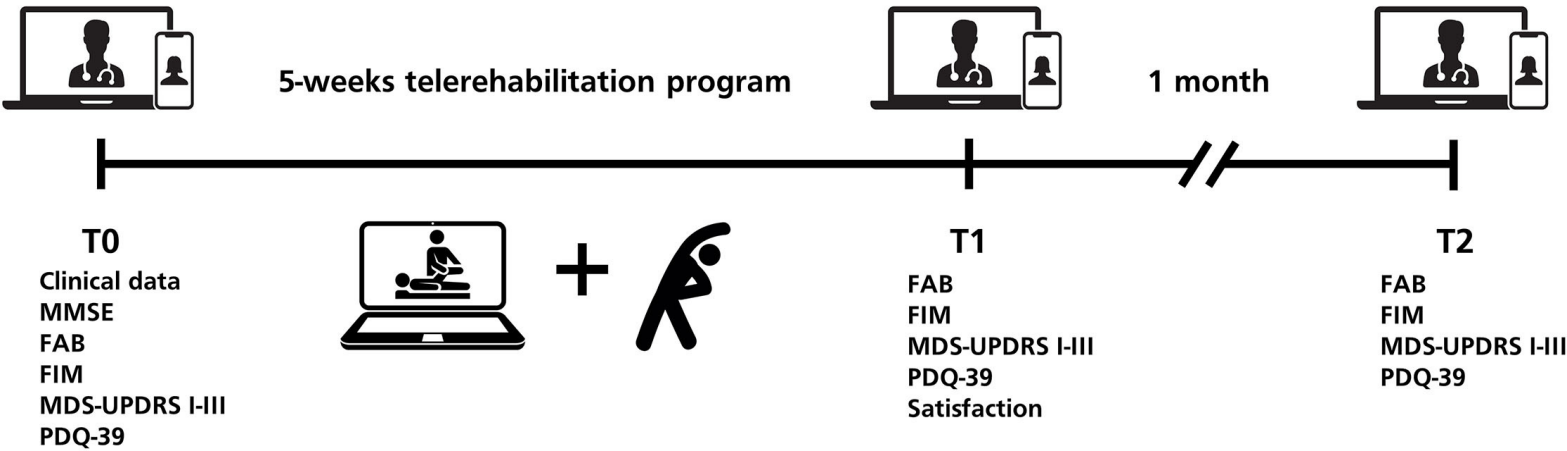
Study design.

The MDS-Unified Parkinson's Disease Rating Scale (MDS-UPDRS) parts I-III were used to assess the motor symptoms severity and the impact of motor and non-motor symptoms on daily life (43). The Parkinson's Disease Questionnaire-39 (PDQ-39) was used to evaluate patients QoL (44). The Functional Independence Measure (FIM) was used to assess functional independence in daily life activities (45). The Frontal Assessment Battery (FAB) was employed to evaluate the frontal cognitive abilities of enrolled patients (46). At the end of the telerehabilitation program, patients were administered a questionnaire composed of five questions constructed as a 7-items Likert scale, investigating the satisfaction for the telerehabilitation program (Q1), the usefulness of the program for PD patients (Q2), the satisfaction for the remote visit modality (Q3) and the willingness to participate again in the same telerehabilitation protocol or other telemedicine programs (Q4 and Q5; Figure 1).

Due to the exploratory nature of the study, a rigorous sample size calculation was not carried out. However, we predicted high compliance for telerehabilitation programs with a low dropout rate. Therefore, we fixed the number of enrolled patients at 25, considering a dropout rate of 20%. All statistical analyses were carried out using the SPSS version 23 software for Windows. The normality of distribution of the variables was assessed using the Shapiro–Wilk test. To assess the effect of the telerehabilitation program across the different time-points on the evaluated variables, repeated measure analysis of variance (rANOVA) was performed. Greenhouse–Geisser correction for non-sphericity and Bonferroni's correction for multiple tests were applied when needed. To evaluate the effect size of our intervention partial η^2^ (η^2^_*p*_) was reported and a *post-hoc* analysis to compute achieved power was performed using G^*^Power software 3.1.9.7 for Windows. The level of significance was set at *p* < 0.05. All data are reported as Mean ± *SD* or Median (Q1–Q3; Min–Max).

## Results

**Figure 2:**
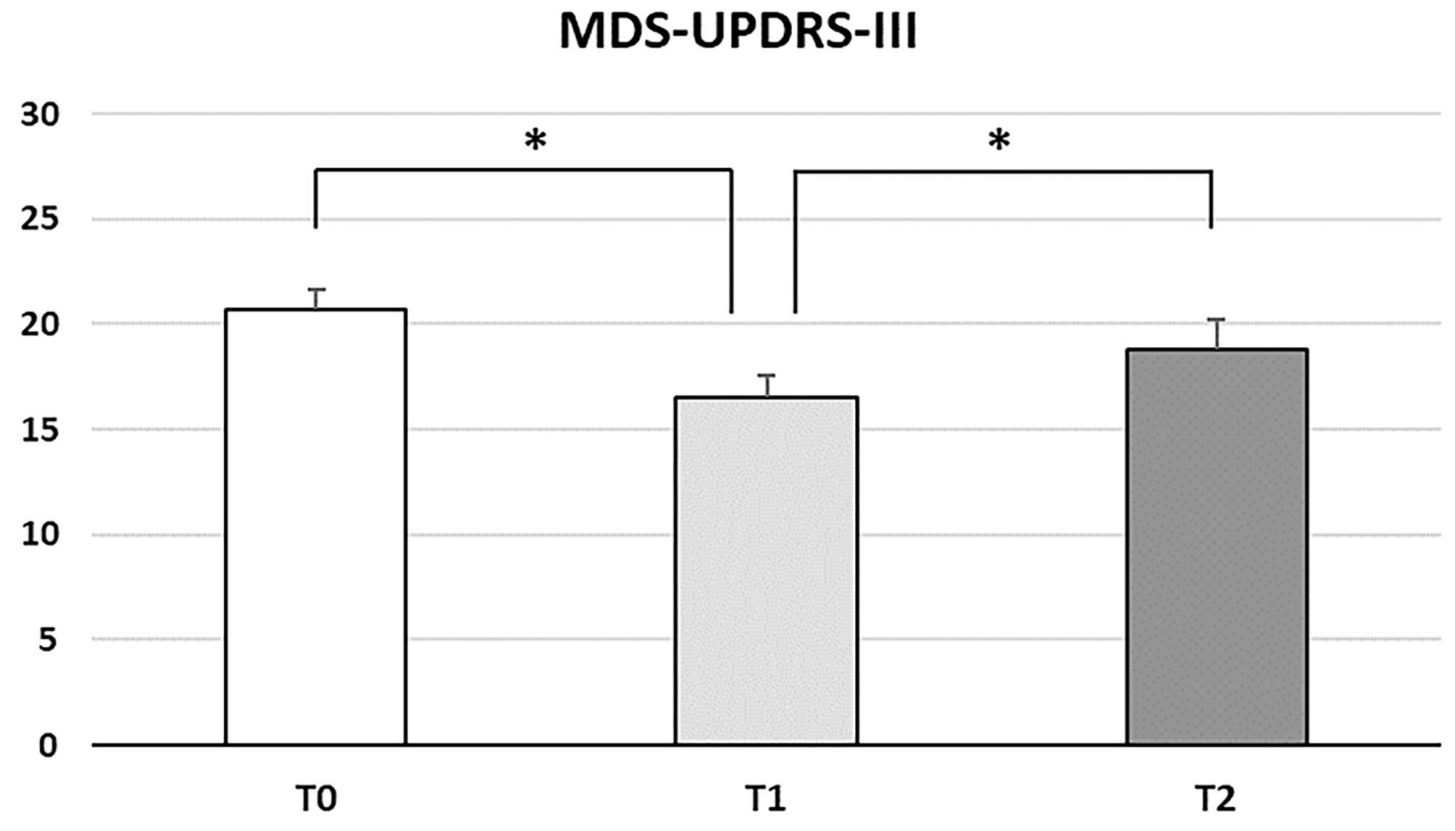
Histogram showing the MDS-UPDRS-III score across time points. Standard error of the mean is shown by vertical bars. Statistically significant differences are marked with an asterisk. The *post-hoc* analysis showed a reduction between T0 and T1 and a return to baseline at T2.

Forty-seven patients were screened for eligibility for the study and 23 (48.9%) were enrolled based on inclusion and exclusion criteria (Supplementary Figure 1). Demographic and clinical features of enrolled patients are shown in Table 1. All patients completed the study, resulting in a dropout rate of 0%. A total of 452 training sessions were completed, 380 of which (83.9%) reached the duration cut-off of 30 min. In 94 out of 115 training weeks (81.7%), the *a priori* criteria of at least 3 sessions/week for minimum 30 min each were reached. When considering single patients, 20/23 (87%) patients reached the cut-off for acceptable adherence of at least 60% of matching frequency and duration, and 16/23 patients (69.6%) reached the optimal cut-off of 80%. No falls or other AEs were reported and no interventions by caregivers were necessary during supervised or self-conducted sessions. Repeated measure ANOVA showed a significant effect of the factor “time” for the MDS-UPDRS-III score across the different time points (*F*_2, 44_ = 10.539; *p* < 0.0001). The *post-hoc* analysis showed a motor severity score significantly reduced right after the treatment with a mean decrease of 4.217 (95% CI, 1.637–6.798; *p* = 0.001), with a return to baseline values at 1-month evaluation (T1 vs. T2 *p* = 0.036; T0 vs. T2 *p* = 0.147; Figure 2). No significant effect of factor “time” was found for the other secondary outcome measures, which remained stable from the beginning to the end of the study. Variables values across time points, the values of η^2^_*p*_ and achieved power are shown in Table 2. Over 90% of patients were “extremely satisfied” or “very satisfied” for the telerehabilitation and remote visit modality and considered the intervention “extremely useful” or “very useful” for PD patient. Furtherly, all except a single patient were highly interested in undergoing again the telerehabilitation program or other telemedicine projects (Supplementary Figure 2).

**Table 1:** Demographic and clinical characteristics of enrolled patients.

**PD patients (*****N*** **=** **23)**		
Age (years)		64.1 ± 8.9
Sex	F	10 (43.5%)
	M	13 (56.5%)
Disease Duration (years)		6.5 ± 3.8
H&Y		2 (2–2; 1–2.5)
MMSE		30 (29–30)
LEDD (mg)		581.5 ± 210.2
Therapy	DA or iMAO-B monotherapy	4 (17.4%)
	L-DOPA monotherapy	3 (13%)
	L-DOPA + Add-on	16 (69.6%)

*DA, dopamine agonist; iMAO-B, MAO-B inhibitors; LEDD, levodopa equivalent daily dose*.

*Variables are shown as Mean ± SD or Median (Q1–Q3; Min–Max) for numerical variables and N (%) for categorical variables*.

**Table 2:** Secondary outcome measures scores at T0–T2.

	**T0**	**T1**	**T2**	**rANOVA**	***p***	**η^2^**_***p***_	**Power%**
MDS-UPDRS-I	8.87 ± 3.98	7.74 ± 3.67	8.17 ± 3.8	F (2, 44) = 2.002	0.147	0.083	39.1%
MDS-UPDRS-II	6.87 ± 4.2	6.3 ± 3.31	6.57 ± 3,68	F (1.559, 34.292) = 0.430	0.604	0.019	10.8%
MDS-UPDRS-III	20.7 ± 4.73	16.48 ± 5.32	18.78 ± 6.75	F (2, 44) = 10.539	<0.0001*	0.324	98.4%
PDQ-39	17.87 ± 10.86	17.26 ± 11.99	15.52 ± 9,96	F (1.424, 31.333) = 1.031	0.345	0.045	18.8%
FIM	122.09 ± 5.25	122.39 ± 4.27	122.35 ± 4.27	F (2, 44)= 0.073	0,929	0,003	6%
FAB	14 (13–15); (7–15)	14 (13–15); (6-15)	14(13-15); (10–15)	F (2, 44) = 1.526	0.229	0.065	30.7%

*For repeated measures ANOVA, F-statistics, effect sizes, and power are reported. Statistically significant results are marked in bold with an asterisk. $\eta_{\text{p}}^{2}$, partial eta squared. Variables are shown as Mean ± SD or Median (Q1–Q3; Min–Max)*.

## Discussion

In this open-label pilot study, we investigated the feasibility, safety, and efficacy of telerehabilitation in mild-to-moderate PD patients. Telemedicine has been applied recently under specific circumstances, for specific indications and eligible patients. Despite the potential relevance of telemedicine for diagnosis, consultation, monitoring and treatment management, availability, and diffusion of telemedicine is still limited by the clinical and sociodemographic features (24, 25). The issue of telerehabilitation in PD has been promoted during the lockdown for COVID-19 pandemics; however, it appears promising for the management of early stages of PD under normal conditions as well. Safety is a major concern to remote physiotherapy, in particular because of the limited possibility of direct intervention by the operator if the case of AEs. Based on the previous reports that 35–90% of PD patients experience at least 1 fall/year, and 2/3 of cases are recurrent fallers (47), the occurrence of falls was the main safety measure in our study. The *a priori* criterion of no falls was matched in our cohort, indicating the high safety of our telerehabilitation program in mild-to-moderate PD patients. Moreover, there was no report of any other AE, in line with the results of previous studies underlying the safety of remote rehabilitation in PD patients (33). Dropout rate and adherence to the program were considered as measures of feasibility. All participants completed the program and the post-training evaluation (dropout rate 0%), confirming that duration and complexity of exercises were accessible to all participants. Despite the potential bias due to lockdown, we would like to point out that participation in our program remained absolute after the reopening of rehabilitation structures as well. The present findings are, therefore, much more promising compared to those of previous studies showing a 20% dropout rate in elderly subjects engaging in a rehabilitation program (48), and confirm the awareness and willingness of PD patients toward rehabilitation. This concept is further supported by the high adherence to the protocol, as almost 85% of patients reached the acceptable cut-off and 70% reached the optimal cut-off for participation. Thus, the present results indicate that telerehabilitation is a feasible, accessible, and likely rewarding intervention in mild-to-moderate PD patients. However, among screened patients, less than half-matched inclusion and exclusion criteria. This at least partially reflects the strict enrollment criteria used in the present studies and must be taken into account when considering the general applicability of remote physiotherapy intervention in PD. Finally, the high rate of satisfaction and willingness to engage in similar programs among our patients demonstrates that PD subjects are interested in the rehabilitation program and can ensure notable compliance and adherence to treatment.

As to motor outcome measures, we found a significant reduction of MDS-UPDRS-III after telerehabilitation. Despite being a secondary outcome measure, *post-hoc* power analysis demonstrated a statistical power >98% with high effect size, confirming the reliability of the finding. Moreover, the previous studies showed a minimum clinical impact for MDS-UPDRS-III between 2.4 and 3.25 (49), thus the score reduction of 4.22 in our study had a clinically significant impact on the patient's motor symptoms severity. In the literature, the efficacy of physiotherapy on motor symptoms is widely demonstrated (7). Moreover, preliminary studies showed efficacy of non-conventional remote administered rehabilitation strategies, including dance or virtual reality training, on motor and non-motor outcomes in PD patients (33). Our study confirms this extended knowledge to the efficacy of remote administered physiotherapy program on motor symptoms of PD, as measured by the MDS-UPDRS-III score. No significant variation was, however, found regarding functional independence, QoL, NMS, and executive cognitive functions in mild-to-moderate PD patients. This lack of significance may depend on several reasons. First, we enrolled PD patients with a modified Hoehn and Yahr score <3. In particular, patients using ambulation aids, with postural instability or reporting FOG were excluded, primarily for safety reasons. Balance and gait disturbances are among the most disabling impairments in PD patients, strongly limiting functional independence and having a strong impact on QoL (4, 47–50). Secondly, the enrolled patients were mostly cognitively stable and patients with significant cognitive impairment were excluded. The previous studies demonstrated an effect of physical exercise on cognitive function and some effect on NMS (7, 51–54), but the relatively good cognitive and NMS status of our patients could have masked the improvement with a roof effect on our secondary outcome measures.

Beyond these considerations, we acknowledge that this exploratory study suffers from limitations due to the open-label and non-controlled design, the small cohort, the relatively good status of our patients, and the remote motor evaluation. Regarding the number of subjects, this was a pilot study, thus a precise sample size calculation was not carried out. However, the *post-hoc* power analysis confirms the reliability of the reported results. Again, the characteristics of enrolled patients could limit the generalizability of our data due to the relatively good functional and cognitive status and a roof effect in outcome measures. Further studies, including intermediate-to-advanced patients with balance and gait disturbances, cognitive impairment and using ambulation aids could help addressing this issue. Finally, the remote motor evaluation could somehow limit the reliability of our data. MDS-UPDRS-III items 3 and 12 (rigidity and postural instability) cannot be performed during remote visits and some evidence showed the reduced validity of tremor assessment when performed through video (55). However, recent studies demonstrated the feasibility and reliability of MDS-UPDRS-III remote administration (22, 55). Thus, we decided remote evaluation of our patients, also to address the difficulties to access medical services during lockdowns and COVID-19 related restrictions. Future studies, implementing remote evaluation instruments, such as wearable devices, could help overcome this limitation.

## Conclusion

Our findings demonstrate that telerehabilitation is safe, feasible, and effective on motor symptoms in mild-to-moderate PD patients. Thus, remote physiotherapy programs could be viable and useful tools to overcome situations with limited access to healthcare services. Further controlled studies with greater sample size, including patients with higher disease severity, cognitive impairment, and implementing remote assessment instruments could help further expand our results.

## Data Availability Statement

The raw data supporting the conclusions of this article will be made available by the authors, without undue reservation.

## Ethics Statement

The studies involving human participants were reviewed and approved by Ethical Committe Sapienza. The patients/participants provided their written informed consent to participate in this study. Written informed consent was obtained from the individual(s) for the publication of any potentially identifiable images or data included in this article.

## Author Contributions

EB, CO, and FP designed the study and wrote the first draft of the manuscript. CO and CM performed the physiotherapy treatment. EB and MA evaluated patients and collected data. EB performed data analyses. DR, PA, AM, and MS reviewed the manuscript draft. All authors contributed to the article and approved the submitted version.

## Conflict of Interest

The authors declare that the research was conducted in the absence of any commercial or financial relationships that could be construed as a potential conflict of interest.

## Publisher's Note

All claims expressed in this article are solely those of the authors and do not necessarily represent those of their affiliated organizations, or those of the publisher, the editors and the reviewers. Any product that may be evaluated in this article, or claim that may be made by its manufacturer, is not guaranteed or endorsed by the publisher.

## Supplementary Material

Supplementary Figure 1 Flow chart of participants trough the study.

Supplementary Figure 2 Results of satisfaction and willingness to re-engage in similar telemedicine programs.

Supplementary Video 1 Video tutorial showing one of the exercises for lying mobility.

Supplementary Video 2 Video tutorial showing one of the exercises for postural transition from lying to sitting position.

Supplementary Video 3 Video tutorial showing one of the exercises combining coordination and dynamic balance.

Supplementary Video 4 Video tutorial showing one of the exercises for facial mobility.
