# Supplementary figures and images for: Feasibility, Safety, and Effectiveness of Telerehabilitation in Mild-to-Moderate Parkinson's Disease
Source: Front Neurol. 2022 Jun 16;13:909197. doi: 10.3389/fneur.2022.909197 (PMC9245570; doi:10.3389/fneur.2022.909197)

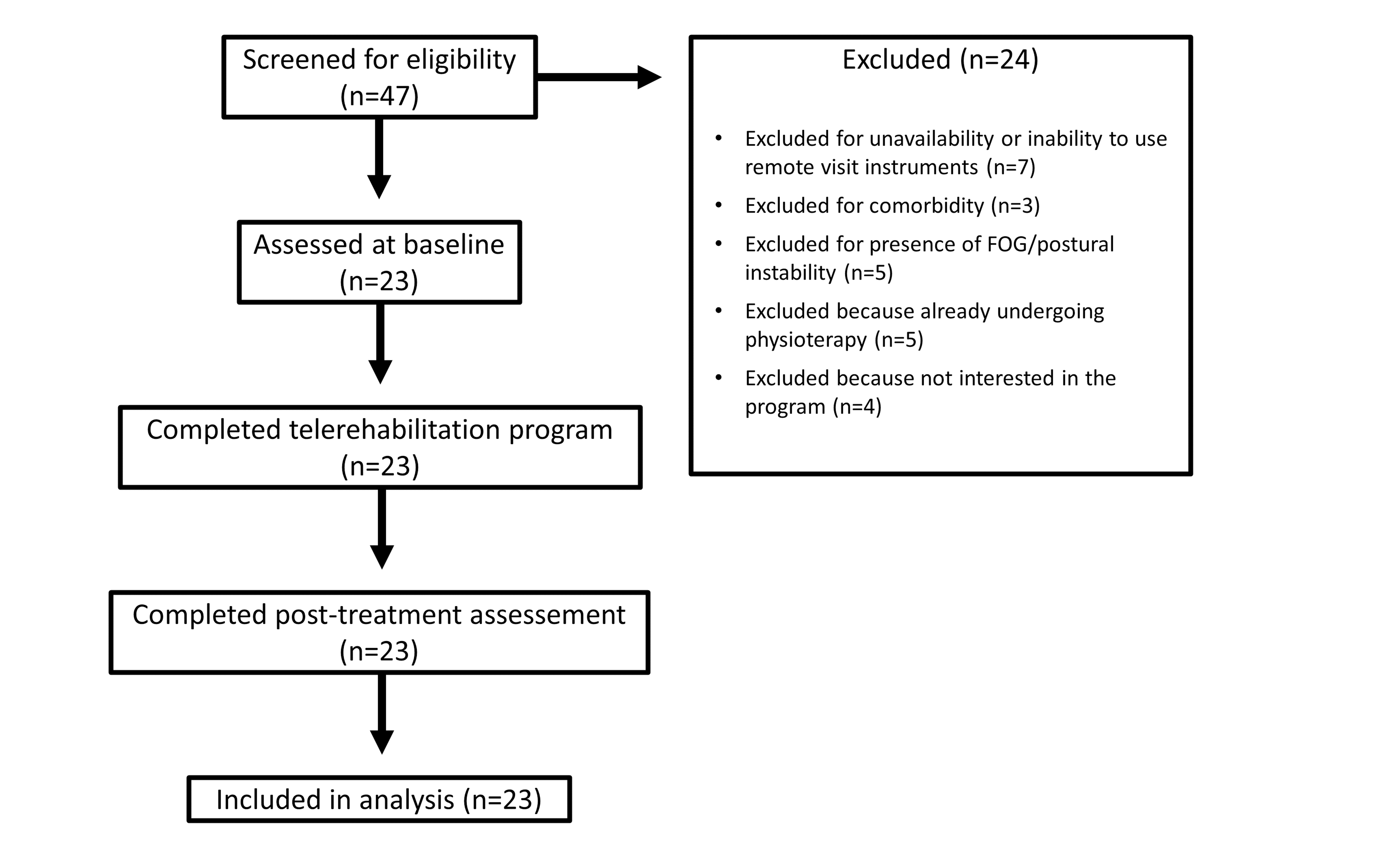

Supplement: Supplementary Figure 1 — Flow chart of participants trough the study. [file Image_1.JPEG]

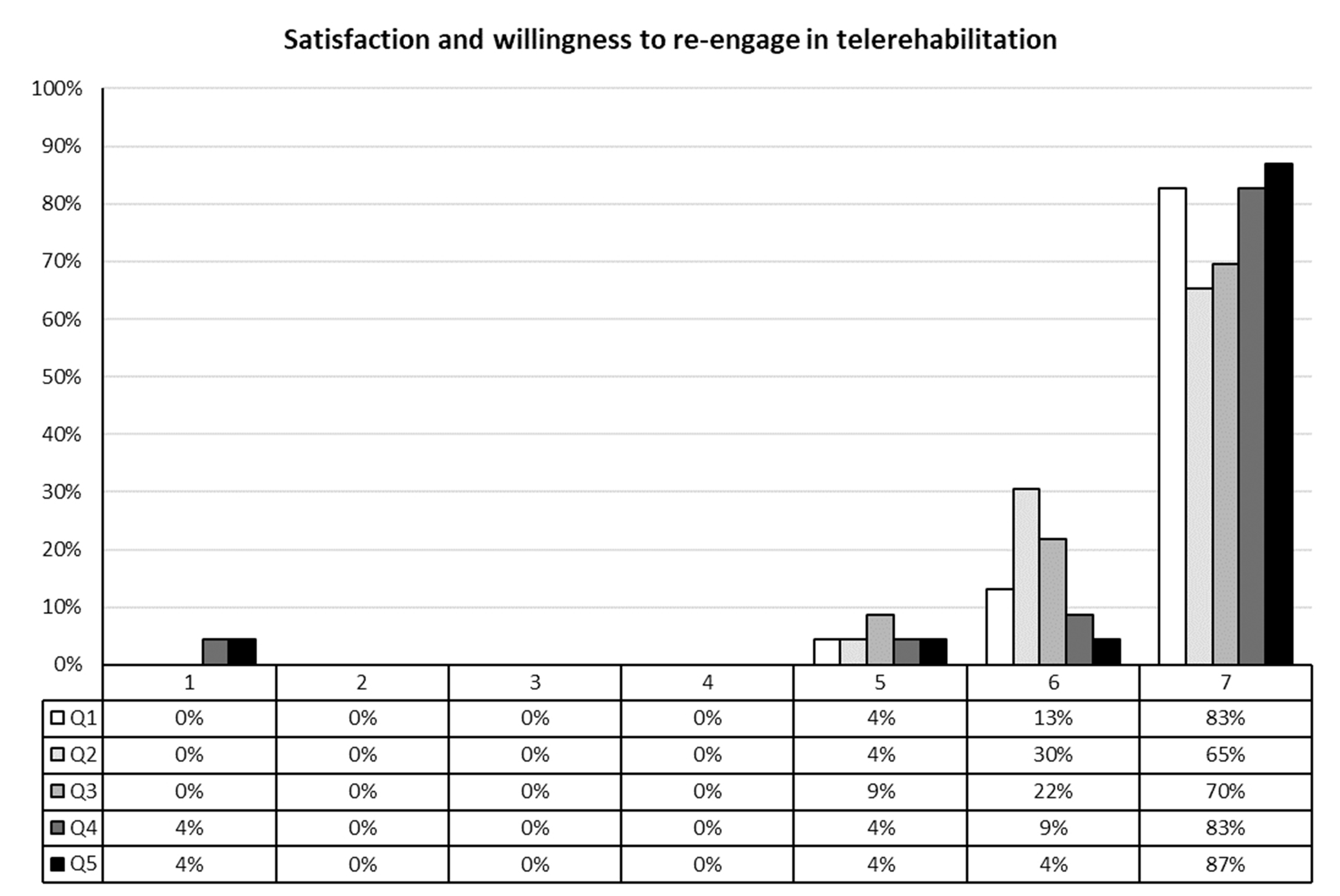

Supplement: Supplementary Figure 2 — Results of satisfaction and willingness to re-engage in similar telemedicine programs. [file Image_2.JPEG]
